# Calligraphy and Painting Identification 3D-CNN Model Based on Hyperspectral Image MNF Dimensionality Reduction

**DOI:** 10.1155/2022/1418814

**Published:** 2022-12-19

**Authors:** Tang XingJia, Zhang PengChang, Xu ZongBen, Hu BingLiang

**Affiliations:** ^1^Institute of Culture and Heritage, Northwestern Polytechnical University, Xi'an 710072, China; ^2^Xi'an Institute of Optics and Precision Mechanics of CAS, Xi'an 710119, China; ^3^School of Mathematics and Statistics, Xi'an JiaoTong University, Xi'an 710149, China

## Abstract

As a kind of cultural art, calligraphy and painting are not only an important part of traditional culture but also has important value of art collection and trade. The existence of forgeries has seriously affected the fair trade, protection, and inheritance of calligraphy and painting. There is an urgent need for the efficient, accurate, and intelligent technical identification method. By combining the advantages of material attribute recognition and imaging detection of hyperspectral imaging technology with the powerful feature expression ability and classification ability of the convolutional neural network, it can greatly improve the comprehension efficiency of calligraphy and painting identification; meanwhile, in order to reduce the redundancy and the amount of parameters in the method of directly using the hyperspectral image, an objective convex dimensionality reduction method should be used for compressing the original hyperspectral image before deep learning. Based on this, we propose a kind of deep learning method to classify author and authenticity based on the multichannel images obtained by minimum noise fraction (MNF) dimensionality reduction to calligraphy and painting hyperspectral data, and its core is the 2D-CNN or 3D-CNN model with the basic network of “4 convolution layers + 4 pooling layers + 2 full-link layers.” The experimental results show that the identification accuracy of the 2D-CNN calligraphy and painting identification with MNF pseudocolor image mosaic as input and the 2D-CNN calligraphy and painting identification with multichannel MNF dimensionality reduced images direct as input have high accuracy, while the 3D-CNN calligraphy and painting identification with multichannel MNF dimensionality reduced images direct as input not only maintains excellent identification accuracy but also has better learning convergence (step number) and stability compared with the 2D-CNN model. Especially, the 3D-CNN identification accuracy of calligraphy and painting's author and authenticity on the test set can reach 93.2% and 95.2%, respectively.

## 1. Introduction

Calligraphy and painting works of art have an important cultural relic value and art transaction value. The existence of fake works seriously affects the fairness and justice of the calligraphy and painting works of art market and the growth of calligraphy and painting culture. The expert eye recognition method is the main appraisal method of discovery false painting now, which uses the spatial visual information of calligraphy and painting, while the information obtained from the eye is the integral accumulated information of visible light, and its spectral resolution is lower. In particular, with the continuous improvement of counterfeiting technology, based on traditional eye recognition, it is difficult to identify high-level counterfeiting means, such as split and high-quality copy. There are a few technical recognition means at present, but their accuracy is limited. Hence there is also an urgent need for better scientific and technological identification means [1–3].

Spectral imaging is an information acquisition mean of attribute recognition and visual perception. For a certain substance, different wavelengths may correspond to different spectral values. Based on this, a relationship curve between wavelengths and spectral values can be drawn. According to the previously mentioned different spectrum curves, substances can be classified. Meanwhile, the spectral image includes the unique differentiation of spectral and spatial characteristics. For calligraphy and painting, although the level of imitation is very high and the spatial information of the fake work is consistent with that of the true one, it is impossible to make the materials completely consistent with the original one. So, there are always some spectral differences in ink, color, paper, chapter, and other aspects, which could be discovered by spectral imaging technology [4].

**Figure 1:**
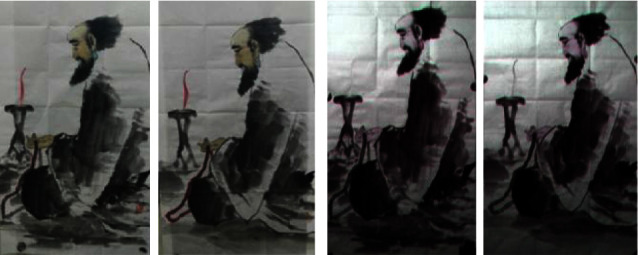
Comparisons of traditional photos and hyperspectral feature images of painting. (a) A true painting's photo and corresponding false painting's photo. (b) True painting's hyperspectral feature image and false painting's hyperspectral feature image.

For example, a comparison between the traditional photos and hyperspectral feature images of true painting and false painting is shown in Figure 1. For the pictures taken by the camera, the wicks of the true work and the fake work are basically the same, but in the hyperspectral feature images consisting of spectrum 930 nm, 780 nm, and 850 nm of the original hyperspectral image, there is some obvious difference; for instance, the right work has the wick, while the left one does not have the wick in the feature images.

Hyperspectral image classification refers to the classification of different date's tags using the spatial features, spectral features, or spatial-spectral joint features of three-dimensional hyperspectral images and includes the supervised and unsupervised ideas. For the pattern recognition and classification based on calligraphy and painting hyperspectral images, the usual method is to use spectral features or spatial features for computing, such as spectral angle matching, support vector machine, particle swarm optimization [5], maximum likelihood, clustering, and decision tree. This kind of method is more suitable for the situation with simple features. For the classification problem with complex features or high-dimensional data, the previous feature analysis method is incomplete or inseparable, which affects the efficiency and accuracy of classification. So, it requires combining the previous methods with data dimensionality reduction, sparse constraints, or finding more efficient classification models, like the method based on locality preserving dimension reduction and Gaussian mixture model [6], the model based on sparse least square support vector machine (LSSVM) [7], the classification by low-rank and sparse representation with adaptive neighborhood regularization [8], the Harris Hawks optimization with principal component analysis (PCA) for dimensionality reduction [9], and so on. Here, in the aspect of hyperspectral image dimensionality reduction, in addition to the aforementioned PCA, it can also be realized by spectrum selection, feature fusion, projection method, and another data transformation [10]. Meanwhile, some shallow networks can also be used to solve the manual feature extraction, such as deep belief network and stack self-encoder network, whose input is a one-dimensional vector, but it is inefficient when it is applied to the feature expression and classification of hyperspectral data [11, 12].

With the development of the intelligent data analysis, the convolution neural network (CNN) becomes a helpful method in machine learning, which realizes the feature expression to high-dimensional data through convolution operation, and significantly reduces the amount of parameters by locally linking effective features and sharing weight theory [13–18].

Generally, CNN mainly has three convolution kernels, which includes one-dimensional convolution (1D-CNN), two-dimensional convolution (2D-CNN), and three-dimensional convolution (3D-CNN). Their network structures are basically similar, but the convolution kernels are different [19–21]. 1D-CNN mainly extracts spectral features and then classifies them without considering spatial features. When they face the phenomena such as “same matter has foreign spectrum” and “foreign matter has same spectrum” in hyperspectral images, it is difficult to obtain good classification results only by spectral information. The most essential difference between 2D-CNN and 1D-CNN is that the convolution and pooling of 2D-CNN are two-dimensional operations. Therefore, 2D-CNN can directly extract the spatial features of hyperspectral images or the dimensionality-reduced data for classification. Whether using characteristic spectral data or dimensionality-reduced images, it mainly uses the target's spatial features, supplemented by some spectral features [22, 23].

There are two main methods for classification based on spatial-spectral joint features in hyperspectral data. One is to reduce the dimension of the hyperspectral data and to extract its two-dimensional features by 2D-CNN firstly, then the 1D-CNN or traditional method is used to extract spectral information; finally, the above spatial features and spectral features are fused to complete the classification. The other is directly using 3D-CNN to extract the spectral-spatial joint features of hyperspectral images. Compared with the spectral or spatial features extracted by 1D-CNN or 2D-CNN, respectively, the features extracted by 3D-CNN are high-dimensional and global features, which are more holistic [24, 25].

It should be mentioned that the calligraphy and painting identification based on hyperspectral images is different from the conventional hyperspectral remote sensing classification. The purpose of traditional hyperspectral remote sensing classification is mainly to classify and recognize different pixels in a single image or multiple images, while the calligraphy and painting recognition based on hyperspectral images is to classify and recognize the labels corresponding to the whole hyperspectral image through the analysis of many hyperspectral images; in other words, the latter is an overall classification. Therefore, the computational complexity of the calligraphy and painting identification based on hyperspectral images is generally higher than that of similar remote sensing image classification. It requires dimensionality reduction of hyperspectral images or parameter reduction of the model in practical learning [26, 27].

To sum up, for the method of using manual features to conduct identification by an expert eye, there are some problems, such as time consumption, laborious manual, and incomplete feature construction. Combining the hyperspectral imaging and machine learning, it can greatly improve the comprehension efficiency of calligraphy and painting identification. Because hyperspectral data could be used for machine learning as a whole without artificial feature extraction, and the different channels of hyperspectral images are redundant, so the calculation complexity of machine learning based on whole hyperspectral images is higher. In order to reduce the redundancy and the amount of network parameters, in this paper, we propose a deep learning method for painting and calligraphy identification based on hyperspectral data, with an objective convex dimensionality reduction for compressing the original hyperspectral image.

## 2. Models of Calligraphy and Painting Identification Based on Hyperspectral Image Dimensionality Reduction

It is supposed that for the hyperspectral image domain of calligraphy and painting, an element can be expressed in an *n*-dimensional space, namely, *x* ∈ **X**(**R**^*n*^), then the painting identification based on hyperspectral dimensionality reduced image is to find a dimensionality reduction mapping function *c*(·) in the previous *n*-dimensional space, and as a result, different kinds of painting can be divided in the mapped dimensionality reduced space. Taking the classification of different calligraphy and painting author as an example, for the calligraphy and painting hyperspectral data of any two authors *x*_*t*1_ ∈ **X**(**R**^*n*^) and *x*_*t*2_ ∈ **X**(**R**^*n*^), there is

(1) $$\begin{array}{c} f\left(c\left(x_{t1}^{i}\right),c\left(x_{t2}^{k}\right)\right)>f\left(c\left(x_{t1}^{i}\right),c\left(x_{t1}^{j}\right)\right)\text{.} \end{array}$$

Here, *x*_*t*1_^*i*^ and *x*_*t*2_^*k*^ represent *i*th sample and *k*th sample in two different author's data sets; *x*_*t*1_^*i*^ and *x*_*t*1_^*j*^ represent *i*th sample and *j*th sample in the same author's data set. In other words, based on the dimensionality reduction mapping *c*(·), the calligraphy and painting in the original *n*-dimensional space would be mapped in a new lower dimensional space; as a result, the mapped data has the smallest measure under the operation of the classification operator *f*(·).

Then, the method of calligraphy and painting identification based on hyperspectral dimensionality reduced images can be divided into two steps: firstly, it is needed to find convex data dimensionality reduction mapping which makes the dimensionality reduced data be one-to-one mapping with the original space. Secondly, the subspace partition function in the dimensionality reduced space is designed, and the partition of the reduced dimension space by the subspace partition function has no intersection, or the intersection is the smallest.

Here, hyperspectral data dimensionality reduction is a projection method in the continuous space, which can be expressed as follows:

(2) $$\begin{array}{c} c\left(x\right)=c_{\left(1,2,\cdots ,s\right)}\left(x\right),c\left(R^{n}\right)⟶R^{s},s\ll n\text{.} \end{array}$$

Among them, any *n*-dimensional data can be projected into a new lower dimensional space by a unified continuous feature map *c*(·), where the new space may not be complete, at least convex.

Hence, the first task in this paper is to find a space dimensionality reduction transformation so as to construct a calligraphy and painting identification model based on the dimension reduced data.

## 3. Calligraphy and Painting Hyperspectral Image Dimensionality Reduction Based on MNF Transformation

For the hyperspectral data dimensionality reduction, it is needed to meet the following conditions: firstly, the feature information of the data is preserved as much as possible; secondly, the data redundancy and correlation should be removed. The mainstream dimensionality reduction methods of hyperspectral data include the method based on the spectral segment selection and the dimensionality reduction method based on data transformation. Among them, segment selection refers to select multiple fewer channel data from the original data, and the original spectral information in the selected multiple channel data is maintained as much as possible. Here, manual selection may inevitably lead to the situation that the dimensionality reduced space and the original space does not meet the one-to-one mapping.

Feature extraction refers to the mathematical transformation of the original data, which is more suitable for dimensionality reduction of high-dimensional data. The dimensionality reduction method-based transformation is to compress the searching space, where the dimensionality reduction mapping is continuous. Common transformation methods include principal component analysis (PCA), minimum noise fraction (MNF) [28], and independent component analysis (ICA). PCA is a commonly used dimensionality reduction method for hyperspectral data. Its essence is to rank according to the variance and to retain several principal components with larger eigenvalues. In fact, only when the noise of original data is independent of the data and the noise variance of all channels data is same, the principal components sorted by variance are consistent with the principal components sorted by noise. Therefore, a dimensionality reduction method which can eliminate the influence of noise is required. MNF is a method of solving the previous problems by a two-layer PCA and selected for hyperspectral dimensionality reduction in the next study, which enable the transformed components to be selected according to the signal-to-noise ratio, so the noise in the transformation component is removed more accurately.

It is assumed that the original calligraphy and painting hyperspectral image **F** is composed of ideal image **F**^0^ and noise image **N** through filter processing, which could be expressed as follows:

(3) $$\begin{array}{c} F=F^{0}+N\text{.} \end{array}$$

Because filtered ideal spectral data and noise data are not related, it is set that the covariance matrices of the ideal image and the noise image are **C**_0_ and **C**_*N*_, so the covariance matrices of original hyperspectral image **F**^0^ can be written as follows:

(4) $$\begin{array}{c} C_{F}=C_{0}+C_{N}\text{.} \end{array}$$

The noise fraction (NF) is defined as the ratio of the noise variance to the total variance of the data; that is, for the linear combination **F***α* of the original spectral data **F**, the noise fraction is as follows:

(5) $$\begin{array}{c} NF=\frac{\alpha^{T}N^{T}N\alpha }{\alpha^{T}F^{T}F\alpha }=\frac{\alpha^{T}C_{N}\alpha }{\alpha^{T}C_{F}\alpha }\text{.} \end{array}$$

Here, *α*=[*α*_1_, *α*_2_, ⋯,*α*_*λ*_]^T^ is the *λ* dimensional linear representation vector.

Similarly, the signal-to-noise ratio (SNR) is defined as the ratio of the variance of filtered spectral data to the variance of noise, that is,

(6) $$\begin{array}{c} SNR=\frac{\alpha^{T}F_{0}^{T}F_{0}\alpha }{\alpha^{T}N^{T}N\alpha }=\frac{\alpha^{T}C_{0}\alpha }{\alpha^{T}C_{N}\alpha }=\frac{\alpha^{T}C_{F}\alpha -\alpha^{T}C_{N}\alpha }{\alpha^{T}C_{N}\alpha }=\frac{1}{NF}-1\text{.} \end{array}$$

It can be seen from equation (6) that the minimum noise fraction (NF) can be obtained by maximizing the signal-to-noise ratio (SNR). That is, the essence of MNF is to maximize the following formula:

(7) $$\begin{array}{c} \frac{1}{NF}=\frac{\alpha^{T}C_{F}\alpha }{\alpha^{T}C_{N}\alpha }\text{.} \end{array}$$

Therefore, the minimum noise transformation (MNF) can be divided into two steps: (1) noise unitization transformation so that *α*^*T*^**C**_*N*_*α* is a unit matrix; (2) spectral principal component transformation is to find the corresponding principal component to *α*^*T*^**C**_*F*_*α*. The specific implementation process of MNF transformation is given as follows.

Noise unitization transformation: the covariance matrix **C**_*N*_ of noise data **N** is obtained by noise estimation; we diagonalize it, and its diagonalization matrix **D**_*N*_ can be expressed as follows:

(8) $$\begin{array}{c} D_{N}=U^{T}C_{N}U\text{.} \end{array}$$

In the formula, **D**_*N*_ is a diagonal matrix consisting of the eigenvalues of **C**_*N*_ in a descending order, and **U** is an orthogonal matrix consisting of the corresponding eigenvectors. Furthermore, it is expressed as follows:

(9) $$\begin{array}{c} E=P^{T}C_{N}P\text{,} \end{array}$$

where **E** is the unity matrix, **P**=**UD**_*N*_^−1/2^. At this time, the spectral data **F** are transformed by the spectral dimension:

(10) $$\begin{array}{c} F^{\prime }=FP=F\left(UD_{N}^{-1/2}\right)\text{.} \end{array}$$

Here, the original spectral data are projected into the transformation space, the transformed data's noise has a unit variance, and the data between different components are not correlated.

Principal component transformation: the covariance matrix **C**_*F*_ of the spectral data **F** is transformed as follows:

(11) $$\begin{array}{c} C_{F-adj}=P^{T}C_{F}P\text{.} \end{array}$$

Then, we diagonalize the prewhitening matrix **C**_*F*−adj_ to obtain the diagonalization matrix **D**_*F*−adj_, which is expressed as follows:

(12) $$\begin{array}{c} D_{F-adj}=V^{T}C_{F-adj}V\text{.} \end{array}$$

In the formula, **D**_*F*−adj_ is a diagonal matrix formed by descending arrangement of eigenvalues of **C**_*F*−adj_, and **V** is an orthogonal matrix formed by corresponding eigenvectors. At this time, the noise unitized spectral transformation data *F*′ that has been transformed in the first step is transformed into the principal component transformation in the second step:

(13) $$\begin{array}{c} I_{mnf}=F^{\prime }V=FPV=F\left(UD_{N}^{-1/2}\right)V\text{.} \end{array}$$

The MNF transformation data **I**_mnf_ of the spectral data **F** are obtained. Here, the dimensionality-reduced component in this method has been obtained without the influence of noise, which is lesser sensitive to noise than the principal component.

Generally, we can obtain the MNF images with the same number of spectral segments. In order to achieve spectral segment compression or dimensionality reduction, the first few transformed images with the largest amount of information are selected as the input data for identification. In practical application, for selecting the appropriate number of MNF channels, the characteristic contribution rate curve under the number of MNF channels is calculated. Here, the calculation formula of the characteristic contribution rate of the first *i* channels of MNF images is expressed as follows:

(14) $$\begin{array}{c} R_{i}=\frac{\sum_{1}^{i}e_{s}}{\sum_{1}^{m}e_{s}}\text{.} \end{array}$$

Here, **e**=[*e*_1_, *e*_2_, ⋯, *e*_*m*_] is the eigenvalue vector of the matrix **D**_*F*_. In engineering application, the characteristic contribution rate of dimensionality reduced data is generally needed to reach 85%.

## 4. Convolution Neural Network Based on Multichannel Dimensionality Reduced Images

Based on the above, the MNF-transformed feature images are still multichannels, but the channel number of feature images with effective information becomes lesser, which make modeling and learning more convenient. In this section, the convolution neural networks based on the previous dimensionality reduced data are discussed under different convolution cores.

### 4.1. Convolution Neural Network Based on Multichannel Dimensionality Reduced Images

**Figure 2:**
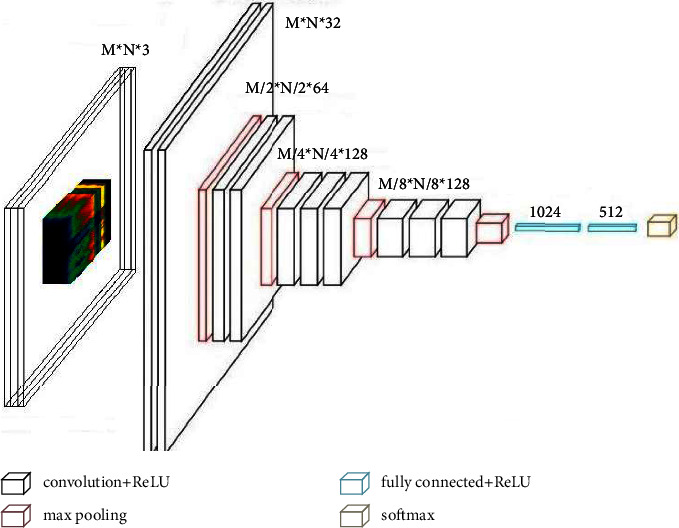
Basic network based on the convolutional neural network for calligraphy and painting identification.

With the in-depth development of machine learning technology, a series of representative networks have been designed. As one of the classic networks, the visual geometry group (VGG) network improves the classification performance to a certain extent by increasing the network depth. The VGG network has two common structures [29, 30], namely, VGG16 and VGG19. Their difference is the different network depth. Compared with the earlier classical network, one improvement of the VGG network is to replace the larger convolution kernel with several consecutive smaller convolution kernels. In this paper, referred to the design of the VGG network, a new depth network is presented as shown in Figure 2. It has the characteristics of equal increase in depth and equal decrease in the width of each layer so as to ensure stable parameter quantity and sufficient complex feature extraction ability in the feature extraction stage. The model mainly includes an input layer, four convolution layers, four pooling layers, two full-link layers, and an output layer. The detailed network design is discussed separately in the following different models.

Assuming that the input data of calligraphy and painting appraisal network are **X** and the output of calligraphy and painting appraisal network is **y**, for the current *i*th layer, **x**_*i*_ is its input, then the input of the *i* + 1th layer can be expressed as follows:

(15) $$\begin{array}{c} x_{i+1}=f_{i}\left(w_{i}^{T}x_{i}+b_{i}\right)\text{.} \end{array}$$

Here, **w**_*i*_ is the weight of the *i*th layer, **b**_*i*_ is the offset, and *f*_*i*_ is the excitation function.

Here, for increasing the nonlinear expression of the network, *ReLU* function is preferentially selected as the excitation function of the convolution layer and full-link layer, whose expression is as follows:

(16) $$\begin{array}{c} ReLU=h\left(w_{i}^{T}x_{i}+b_{i},0\right)=\left\{\begin{array}{c} w_{i}^{T}x_{i}+b_{i},\;w_{i}^{T}x_{i}+b_{i}>0\\ 0,\;else \end{array}\right.\text{.} \end{array}$$

Furthermore, for reducing the parameters and feature dimension, max pooling is preferred; meanwhile, softmax function is adopted in the output layer.

At this time, the network's output predicts the possibility belonging to each class in the current iteration. The specific calculation formula is as follows:

(17) $$\begin{array}{c} p_{w}\left(x^{\left(t\right)}\right)=\left[\begin{array}{c} p\left(y^{\left(t\right)}=1\left|x^{\left(t\right)}\right.;w\right)\\ p\left(y^{\left(t\right)}=2\left|x^{\left(t\right)}\right.;w\right)\\ \cdots \\ p\left(y^{\left(t\right)}=k\left|x^{\left(t\right)}\right.;w\right) \end{array}\right]=\frac{1}{\sum_{j=1}^{k}e^{w_{j}^{T}x^{\left(t\right)}}}\left[\begin{array}{c} e^{w_{1}^{T}x^{\left(t\right)}}\\ e^{w_{2}^{T}x^{\left(t\right)}}\\ \ldots \\ e^{w_{k}^{T}x^{\left(t\right)}} \end{array}\right]\text{,} \end{array}$$

where **w**=[**w**_1_^*T*^; **w**_2_^*T*^; ⋯, **w**_*k*_^*T*^] represents all model parameters. The function of operation 1/∑_*j*=1_^*k*^*e*^**w**_*j*_^*T*^**x**^(*t*)^^ is to do normalization so that the sum of all classes' probability is 1.

The training process of CNN is mainly divided into two parts: forward propagation and backward propagation. Backward propagation is used to update the training parameters to minimize the difference between the current classification results and the target classification, which is defined as follows:

(18) $$\begin{array}{c} J\left(w\right)=-\frac{1}{m}\overset{m}{\underset{t=1}{\sum }}\overset{k}{\underset{j=1}{\sum }}1\left\{y^{\left(t\right)}=j\right\}\log\left(p_{j}^{\left(t\right)}\right)\text{,} \end{array}$$

where *m* is the number of training samples, **y** is the target output and *p*_*j*_^(*t*)^ is the probability value of predicting that the *t*th training sample **x**^(*t*)^ belongs to class *j*:

(19) $$\begin{array}{c} p\left(y^{\left(t\right)}=j\left|x^{\left(t\right)}\right.;w\right)=\frac{e^{w_{j}^{T}x^{\left(t\right)}}}{\overset{k}{\underset{l=1}{\sum }}e^{w_{l}^{T}x^{\left(t\right)}}}\text{.} \end{array}$$

During forward propagation, the previous formulas will be used to calculate the classification results through the current network parameters. The formula 1{**y**^(*t*)^=*j*} indicates that it is 1 if class *j* is the same to real label, otherwise it is 0.

In the network training, the gradient descent algorithm is used to update the parameters. It is needed to calculate the gradient of the price function *J*(**w**):

(20) $$\begin{array}{c} \nabla_{w}J\left(w\right)=-\frac{1}{m}\overset{m}{\underset{i=1}{\sum }}\left[x^{\left(t\right)}\left(1\left\{y^{\left(t\right)}=j\right\}-p\left(y^{\left(t\right)}=j\left|x^{\left(t\right)}\right.;w\right)\right)\right]\text{.} \end{array}$$

Then, the parameter iteration is carried out through the formula **w**=**w** − *α* · ∇_**w**_*J*(**w**), where *α* is the learning rate.

### 4.2. 2D-CNN Model Based on Multichannel Dimensionality Reduced Image

The 2D-CNN model based on multichannel dimensionality reduced image is also composed of the input layer, convolution layer, pooling layer, full-link layer, and output layer. Its characteristic is that the multiple dimensional data would be respectively convoluted and then summed; the following is the specific description.

**Figure 3:**
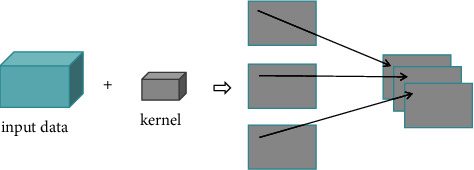
Schematic diagram of 2D-CNN convolution to process high-dimensional data.

Figure 3 shows that in the 2D-CNN convolution layer, the convolution core moves in two spatial directions. When the input is a color image, the input of the 2D-CNN model is three channels. If we want to use it for hyperspectral images or multichannel dimensionality reduced images of hyperspectral images, we can change the channel number from 3 to a greater value. Here, it is set that the channel number of calligraphy and painting multichannel dimensionality reduced images is *m*, the size of input image can be expressed as (*m*, *h*, *w*), and the size of convolution kernel is (*m*, *P*, *Q*). At this time, the convolution kernel is performed to do sliding window convolution operation of all values in a window (*P*, *Q*) on *m* channels. When all the sliding window calculations are completed, the calculated values in different channels are summed to obtain a feature image.

For the high-dimensional image, one 2-dimensional convolution kernel could be used to get one feature image, which means that the weighted product of different channel images to one convolution kernel is finished for obtaining one feature image. When *n* convolution kernels are used, a group of *n*-dimensional feature images can be obtained. At this time, the calculation formula of the convolution layer can be expressed as follows:

(21) $$\begin{array}{c} v_{i,j}^{x,y}=f\left(\overset{m}{\underset{s=1}{\sum }}\overset{P_{i}-1}{\underset{p=0}{\sum }}\overset{Q_{i}-1}{\underset{q=0}{\sum }}w_{i,j,s}^{p,q}v_{i-1,s}^{x+p,y+q}+b_{i,j}\right)\text{,} \end{array}$$

where *v*_*i*,*j*_^*x*,*y*^ represents the value of the neuron at the position (*x*, *y*) in the *j*th feature image of the *i*th layer; *f* is the activation function, and the height and width of the convolution kernel are expressed by *P*_*i*_ and *Q*_*i*_, respectively; the weight of the *s*th feature image of *i* − 1th layer linked to the neuron at the position (*p*, *q*) in the *j*th feature image of the *i*th layer is *w*_*i*,*j*,*s*_^*p*,*q*^, which is weighted, produced, and summed with the value of the position (*x*+*p*, *y*+*q*) in the *s*th feature image of the *i* − 1th layer, and *b*_*i*,*j*_ indicates the bias.

### 4.3. 3D-CNN Model Based on Multichannel Dimensionality Reduced Image

The input of the 3D-CNN model is high-dimensional data, the convolution kernel is all high-dimensional, and it can extract features in three directions. So, for hyperspectral images or hyperspectral dimensionality reduced images, it is processed by the convolution kernel in three directions; it is no longer simply to extract spatial features or spectral features but to capture the overall spatial and spectral features as a whole.

Similar to the 2D-CNN model, the 3D-CNN model is composed of the input layer, 3D convolution layer, 3D pooling layer, full-link layer, and output layer. Unlike 2D-CNN, the characteristic of 3D-CNN is that the multiple dimensional data would be convoluted but not summed; the following is the further discussion.

**Figure 4:**
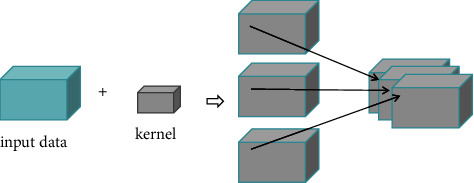
Schematic diagram of 3D-CNN convolution to process high-dimensional data.

It is supposed that the size of the input data is (*m*, *h*, *w*), the channel number of the convolution kernel is *n*, and the size of the convolution kernel is (*n*, *P*, *Q*, *R*).  Figure 4 shows that it is the convolution layer of the 3D-CNN convolution model.

It can be seen that the 3D convolution operation is no longer to process multiple channels of data such as the 2D convolution kernel but to do weighted product in multiple dimensional data directly without summation. In this structure, each feature in the convolution layer will be connected with adjacent multiple dimensional data in the upper layer, and a 3D feature is obtained through 3D sliding window convolution operation. Therefore, it has the better overall expression ability of spatial-spectral features.

At this time, the calculation formula of 3D convolution is

(22) $$\begin{array}{c} v_{i,j}^{x,y,z}=f\left(\overset{n}{\underset{s=1}{\sum }}\overset{P_{i}-1}{\underset{p=0}{\sum }}\overset{Q_{i}-1}{\underset{q=0}{\sum }}\overset{R_{i}-1}{\underset{r=0}{\sum }}w_{i,j,s}^{x,y,r}v_{i-1,s}^{x+p,y+q,z+r}+b_{i,j}\right)\text{,} \end{array}$$

wherein *v*_*i*,*j*_^*x*,*y*,*z*^ represents the value of the neuron at the position (*x*, *y*, *z*) in the *j*th feature data of the *i*th layer; *f* is the activation function, and the length, height, and width of the convolution kernel are expressed as *P*_*i*_, *Q*_*i*_, and *R*_*i*_, respectively; the weight of the *s*th channel linked to the neuron at the position (*x*, *y*, *z*) in the *j*th feature data of the *i*th layer is *w*_*i*,*j*,*s*_^*p*,*q*,*r*^, which is weighted, produced, and summed with the *s*th feature data at the position (*x*+*p*, *y*+*q*, *z*+*r*) of *i* − 1th layer; *b*_*i*,*j*_ indicates the bias.

In addition, the pooling layer of the 3D-CNN model is also different from the 2D-CNN model, which is realized by using 3D down sampling calculation, such as common 3D max pooling and 3D mean pooling.

## 5. Calligraphy and Painting Identification CNN Model Based on Multichannel Dimensionality Reduced Images

Based on the foregoing, by comparing different model's design and corresponding analysis, a basic network consisting of “4 convolution layers + 4 pooling layers + 2 full-link layers” is obtained. In this section, the different types of convolution kernels and different input design of calligraphy and painting identification CNN model will be discussed firstly, and then the optimal calligraphy and painting identification CNN model will be selected through the experimental comparison in the next section.

### 5.1. 2D-CNN Calligraphy and Painting Identification Model under Multiple MNF Pseudocolor Images of Mosaic as Input

For the calligraphy and painting identification based on multichannel dimensionality reduced images by a single 2D-CNN, considering the difference of information contained in the different channel, it is planned to splice multiple groups of calligraphy and painting MNF pseudocolor images and take them into a single 2D-CNN. In this paper, MNF pseudocolor images consisting of different channel MNF images **I**_mnf(*i*, *j*, *k*)_ are normalized, and then we do mosaic together in the column direction to obtain the new integrated data, which is conveniently used to be the input of the 2D-CNN model, that is,

(23) $$\begin{array}{c} I=concat\left(one\left(I_{mnf\left(i,j,k\right)}\right),2\right)\text{,} \end{array}$$

where concat represents the matrix stitching of multiple matrixes, one represents normalization, and 2 represents the column stitching.

In the application, the specific model design is as follows: the first convolution layer uses the 5 × 5 convolution kernel to generate 32 feature images, the second convolution layer uses the 5 × 5 convolution kernel to generate 64 feature images, the third convolution layer uses the 3 × 3 convolution kernel to generate 128 feature images, and the fourth convolution layer uses the 3 × 3 convolution kernel to generate 128 feature images. All convolution layers are processed by complementing 0, and the length and width of all convolution layers remain unchanged. The pooling layer uses 2 × 2 window for maximum pool operation. The step size of the convolution layer is (1, 1), and the step size of the pooling layer is (2, 2). The two full-link layers adopt 1024 and 512 dimensions, respectively, and the output layer is trained by the classification of calligraphy and painting authors and the identification of calligraphy and painting authenticity, respectively. Here, the classification of calligraphy and painting authors refers to judging the author of calligraphy and painting through data analysis, and the authenticity identification of calligraphy and painting refers to judging whether calligraphy and painting is true or false through data analysis.

### 5.2. 2D-CNN Calligraphy and Painting Identification Model under Multichannel MNF Dimensionality Reduced Images Directly as Input

For the case of using a single 2D-CNN for calligraphy and painting identification based on multichannel MNF dimensionality reduced images, the multichannel MNF dimensionality reduced images **I**_mnf_ could also be directly input into the 2D-CNN model as a whole, instead of using MNF pseudocolor images of mosaic as input.

Similarly, we continue to select the previous unified basic network of “4 convolution layers + 4 pooling layers + 2 full-link layers,” and the specific model design is consistent with the previous model.

### 5.3. 3D-CNN Calligraphy and Painting Identification Model under Multichannel MNF Dimensionality Reduced Images Directly as Input

In this section, the 3D-CNN model is directly used for modeling and learning. In the case of multichannel MNF dimensionality reduced images directly as input, 3D-CNN convolution kernel is used for convolution operation so as to use the high-dimensional feature data. For the convenience of comparison with the previous models based on 2D-CNN, the designation about 3D-CNN in this section is also composed of the input layer, four 3D convolution layers, four 3D pooling layers, two full-link layers, and the output layer; the convolution layer and pooling layer are connected alternately.

The 3D-CNN calligraphy and painting identification model based on multichannel MNF dimensionality reduced images is designed as follows: the first convolution layer uses the 5 × 5 × 3 convolution kernel to generate 9 feature cubes, the second convolution layer uses the 5 × 5 × 2 convolution kernel to generate 16 feature cubes, the third convolution layer uses the 3 × 3 × 1 convolution kernel to generate 32 feature cubes, and the fourth convolution layer uses the 3 × 3 × 1 convolution kernel to generate 32 feature cubes. All convolution layers adopt complement 0, and the length, width, and depth of all convolution layers remain unchanged. The maximum pooling method is used for down sampling by 2 × 2 × 2 window on the three directions. At this time, the step size of the convolution layer is (1, 1, 1), the size of the feature cube decays by 1/2 layer by layer, and the step size of pooling layer is (2, 2, 2). The two full-link layers adopt 1024 and 512 dimensions, and the output layer is trained according to the classification of authors and the identification of authenticity, respectively.

Compared with 2D-CNN, the core of 3D-CNN is to use 3D convolution kernel to perform convolution operation in three directions of hyperspectral images. Not only the spatial structure characteristics of calligraphy and painting can be learned but also the internal correlation characteristics between spectral information with spatial information can be learned through 3D convolution operation. The previous 3D convolution operation of spatial information and spectral information combination gives consideration to both the visual analysis of calligraphy and painting and spectral attribute learning. Meanwhile, the characteristic data after convolution will be connected with several adjacent subgraphs in the upper layer, so 3D-CNN learning is transitive. Hence, the 3D-CNN model can be used in calligraphy and painting identification model based on hyperspectral images.

## 6. Verification Test

In order to test the effect of the previous three kinds of CNN models, the following experiment of calligraphy and painting identification will be finished for selecting the optimal one.

**Figure 5:**
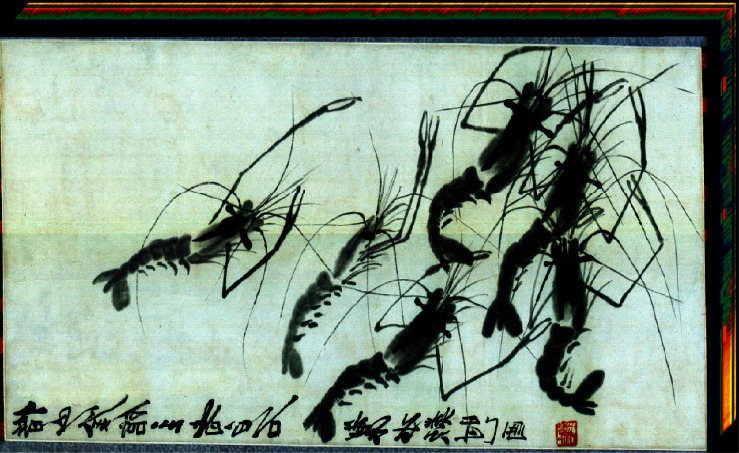
Calligraphy and painting hyperspectral data obtained by the hyperspectral imaging push-scanning system.

Figure 5 shows that the hyperspectral image of a group of Chinese calligraphy and painting are obtained by the calligraphy and painting hyperspectral imaging scanning system. Furthermore, we take Qi Baishi's calligraphy and painting and Lu Yanshao's calligraphy and painting to get the data set. Then, the visible and near-infrared hyperspectral data of Qi Baishi's true painting, Qi Baishi's fake painting, and Lu Yanshao's true painting are collected. Here, the number of spectral channels of visible near-infrared hyperspectral data is 134.

**Figure 6:**
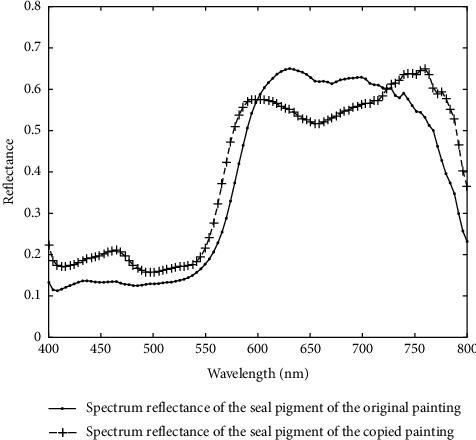
Spectrum of red seals of Qi Baishi's true painting and Qi Baishi's copied painting.

Through data extraction, it is found that the spectrum of red seal of Qi Baishi's true painting is different from the spectrum of red seal of Qi Baishi's copied painting, as shown in Figure 6. In specific experiments, the original hyperspectral images of calligraphy and painting are cut to image block by spatial dimension for getting more data.

### 6.1. MNF Dimensionality Reduction Test for Data Preprocessing

**Figure 7:**
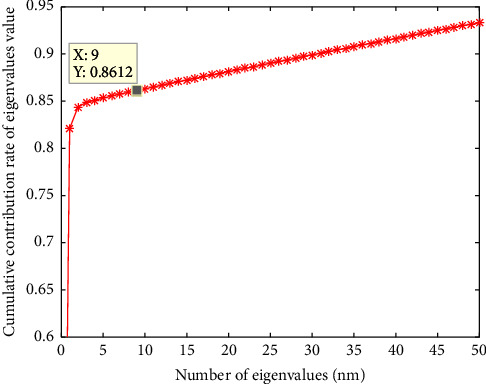
MNF image's characteristic contribution rate curve.

According to the previous designation, the eigenvalue contribution rate is used as basis to select the channel number of MNF images. Generally, we select the appropriate channel number of feature images with 85% as the standard.  Figure 7 shows that it is the eigenvalue contribution rate curve of MNF images of a group of calligraphy and painting hyperspectral images. It can be seen that when the number of characteristic components reaches 5, the main information contribution rate reaches 85%, and with the increase of the number of characteristics, the contribution rate basically increases equivalently and steadily. As mentioned earlier, the network input includes pseudocolor images and multichannel images. Because a pseudocolor image needs three channels and the characteristic contribution rate of the first five channels of the MNF image just exceeds 85%, so it is at least two groups of pseudocolor images, which could satisfy 85% of the contribution rate. In order to ensure that the information amount of the MNF image is as much as possible, another group of pseudocolor images is added in this study. Finally, the channel number of MNF images under dimensionality reduction is 9, namely, the first nine channels of MNF images are selected as the dimensionality reduced data for learning. At this time, the main information contribution rate reaches 86%.

In addition, the size of the input data is compressed to 100 × 100 for meeting the input requirement of the model. After compression, the input data's dimension of 2D-CNN calligraphy and painting identification model under MNF pseudocolor image mosaic as input is 100 × 100 × 3, where the pseudocolor image is similar to the traditional RGB color image. Their difference is that the selected three channels are used to replace the RGB three channels for image display. The input data's dimension of 2D-CNN calligraphy and painting identification model under multichannel MNF dimensionality reduced images directly as input is 100 × 100 × 9, and the input data's dimension of 3D-CNN calligraphy and painting identification model based on multichannel MNF dimensionality reduced images is 100 × 100 × 9.

### 6.2. Calligraphy and Painting Feature Extraction Based on 2D-CNN

**Figure 8:**
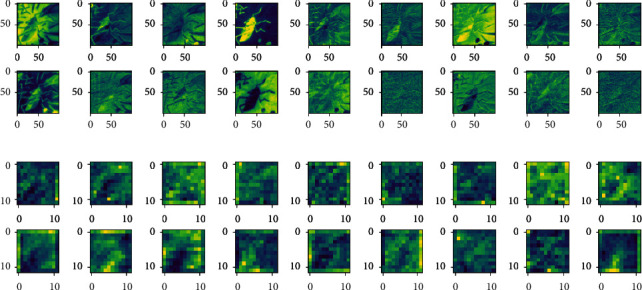
Output comparison of the convolution layer of the 2D-CNN authenticity identification model. (a) Input layer: first nine channels of MNF images (Qi Baishi's true painting in the upper figure/Qi Baishi's fake painting in the following figure). (b) Output feature image of the fourth convolution layer (Qi Baishi's true painting in the upper figure/Qi Baishi's fake painting in the following figure).

In order to evaluate the calligraphy and painting feature extraction ability of 2D-CNN, 9-channel MNF dimensionality reduced image block of Qi Baishi's true painting and 9-channel MNF dimensionality reduced image block of Qi Baishi's fake painting are selected for comparison. The trained 2D-CNN models are used to extract the feature of different input sources. Firstly, for a group of Qi Baishi's true and false calligraphy and painting image block, the 2D-CNN calligraphy and painting authenticity identification model under the multichannel MNF dimensionality reduced image block directly as input is used for feature extraction. At this time, the input image and output of the fourth convolution layer are shown in Figure 8.

**Figure 9:**
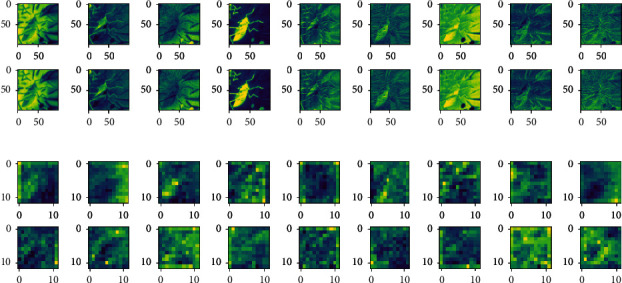
Output comparison of the convolution layer of 2D-CNN models with different functions under the same painting data as input. (a) Input layer: first nine channels of MNF images (the upper shows the 2D-CNN author classification result/the following shows the 2D-CNN authenticity identification result). (b) Output feature images of the fourth convolution layer (the upper shows the 2D-CNN author classification result/the following shows the 2D-CNN authenticity identification result).

It can be seen from the previous explanation that the MNF feature images of the same author's true painting and false painting have shown some differences; especially, when the feature extraction process is carried out by using the 2D-CNN authenticity identification model, with the increase of the depth of the convolution layer, the feature images of Qi Baishi's true and false paintings also show more feature differences. Furthermore, in order to analyze the feature expression ability of 2D-CNN models with different functions on the same input, the author classification model based on 2D-CNN and the authenticity identification model based on 2D-CNN are used to convolute Qi Baishi's same calligraphy and painting data, and then the difference of feature expression is compared. The results are shown in Figure 9.

It can be seen that when the same painting and calligraphy data are input into different 2D-CNN models with different functions, their output features show some difference; with the increase of depth of the convolution layer, it is indicated that the parameters of the feature expression for different output targets are different, and the results of feature expression are also different on texture and color.

### 6.3. Calligraphy and Painting Feature Extraction Based on 3D-CNN

In order to evaluate the feature extraction ability of 3D-CNN for calligraphy and painting data, 9-channel MNF dimensionality reduced the image block of Qi Baishi's true paintings and 9-channel MNF dimensionality reduced the image block of Qi Baishi's fake paintings are selected to feature extraction experiment. At this time, a different feature is extracted by 3D-CNN convolution operation with trained network parameters. Since the features obtained by the 3D-CNN model are 9-channel feature data, in order to facilitate display, only three channel images of each feature data are taken for pseudocolor display.

**Figure 10:**
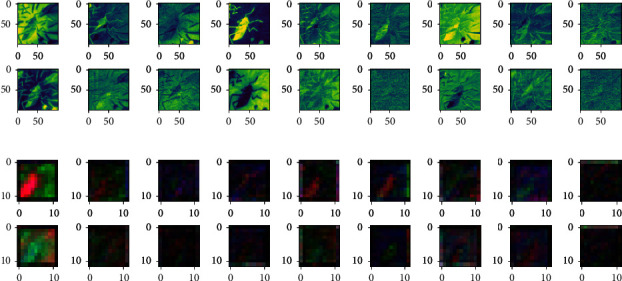
Out comparison of the convolution layer of the 3D-CNN authenticity identification model. (a) Input layer: first nine channels of MNF images (Qi Baishi's true painting in the upper figure/Qi Baishi's fake painting in the following figure). (b) Output feature images of the fourth convolution layer (Qi Baishi's true painting in the upper figure/Qi Baishi's fake painting in the following figure).

Firstly, for the Qi Baishi's true painting and false painting, the 3D-CNN calligraphy and painting authenticity identification model under multichannel MNF dimensionality reduced image block directly as input is used for feature extraction. At this time, the original image and the output features of the fourth convolution layer are shown in Figure 10.

It can be seen from the previous explanation that the MNF feature images of true paintings and false paintings of the same author have shown some differences. The feature extracted by 3D-CNN is a stereo feature, and each feature contains multiple channels; while the feature extracted by 2D-CNN is a two-dimensional feature which contains only one channel; as a result, with the increase of the convolution layer's depth, the feature images of Qi Baishi's true paintings and false paintings carried out by the 3D-CNN authenticity identification model show more differences.

**Figure 11:**
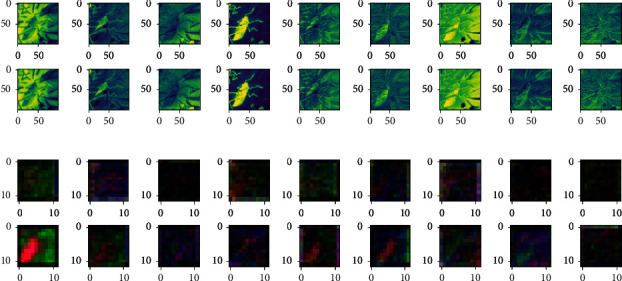
Output comparison of the convolution layer of different functional 3D-CNN networks with the same painting and calligraphy data as input. (a) Input layer: first nine channels of MNF images (the previous figure shows the 3D-CNN author classification result/the following figure shows the 3D-CNN authenticity identification result). (b) Output feature images of the fourth convolution layer (the previous figure shows the 3D-CNN author classification result/the following figure shows the 3D-CNN authenticity identification result).

Similarly, in order to analyze the feature expression ability of 3D-CNN models with different functions when the input image is same, the author classification model based on 3D-CNN and the authenticity identification model based on 3D-CNN are used to convolute a group of Qi Baishi's calligraphy and painting data, and the differences of feature expression are compared in Figure 11.

It can be seen that when the same painting and calligraphy data are input into different 3D-CNN models with different functions, the output feature show some differences. With the increase of convolution layer's depth, it is indicated that the parameters of feature expression are different, and the results of feature expression are also different in texture and so-called color.

In addition, it can be seen from the figure that for Qi Baishi's true painting image blocks and false painting image blocks, the feature expression ability of the 2D-CNN model and 3D-CNN model under the same input is different. Especially, the feature difference of convolution layer's output of the 2D-CNN calligraphy and painting authenticity identification model is obvious in the spatial feature, while the convolution layer's output feature of the calligraphy and painting authenticity identification model based on 3D-CNN is a multidimensional image, which can provide more information in depth of field dimensions. In other words, its feature expression is -multidimensional, and the feature information is richer.

### 6.4. Calligraphy and Painting Identification Experiment Based on Multichannel MNF Dimensionality Reduced Images

In this experiment, we meanly test the efficiency of 2D-CNN and 3D-CNN calligraphy and painting identification models based on multichannel MNF dimensionality reduced images.

#### 6.4.1. Classification Experiment of Different Calligraphers and Painters

In the test, nearly 100 typical calligraphy and painting works of Qi Baishi and 50 typical calligraphy and painting works of Lu Yanshao are used for hyperspectral imaging scanning for obtaining their visible near-infrared hyperspectral data. Because it is difficult to collect the calligraphy and painting samples with similar labels, there are low number of hyperspectral images of calligraphy and painting in the real experiment, so the original spectral images are cut to obtain the hyperspectral image blocks, which are actually used for learning. At this time, the amount of training data is greatly increased to thousands.

**Figure 12:**
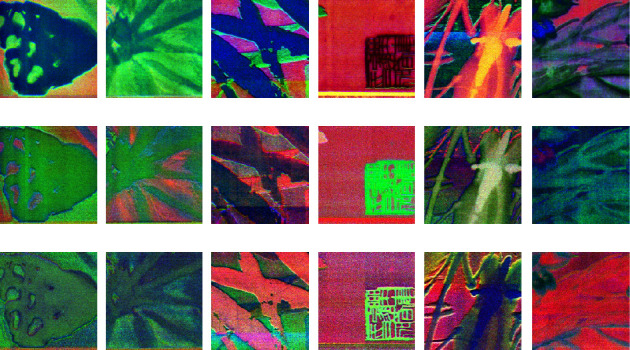
Pseudocolor image blocks were composed of different channel MNF images of Qi Baishi's painting. (a) Qi Baishi's pseudocolor image blocks were composed of 1/4/7-th MNF channel images. (b) Qi Baishi's pseudocolor image blocks were composed of 2/5/8-th MNF channel images. (c) Qi Baishi's pseudocolor image blocks were composed of 3/6/9-th MNF channel images.

**Figure 13:**
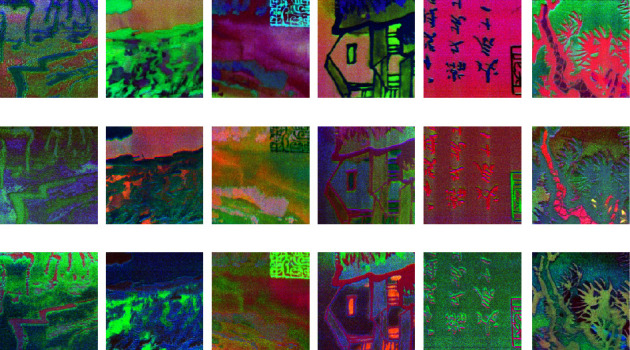
Pseudocolor image blocks were composed of different channel MNF images of Lu Yanshao's paintings. (a) Lu Yanshao's pseudocolor image blocks were composed of 1/4/7-th MNF channel images. (b) Lu Yanshao's pseudocolor image blocks were composed of 2/5/8-th MNF channel images. (c) Lu Yanshao's pseudocolor image blocks were composed of 3/6/9-th MNF channel images.

Then, MNF is carried out to obtain multichannel MNF dimensionality reduced images. The first nine channels of MNF images are taken as learning data, and then the training of different models is carried out according to previous designation. Figures 12 and 13 show that it is a group of pseudocolor image blocks composed of different channel MNF feature images with different authors.

It can be seen that the visual information in MNF pseudocolor images synthesized by different channels MNF feature image is different, which can provide a lot of classification information for learning. There is also some different characteristic in spatial information between Qi Baishi's calligraphy and painting and Lu Yanshao's calligraphy and painting. For example, it focused on ink rendering with more lines about Qi Baishi's calligraphy and painting, while more curves and lines with richer rendering are used in Lu Yanshao's calligraphy and painting.

**Figure 14:**
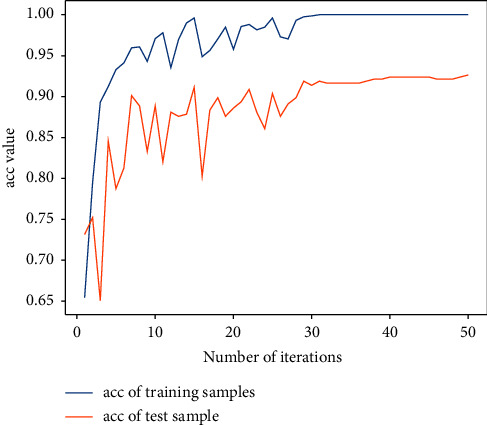
Author identification of the 2D-CNN model under MNF pseudocolor image mosaic as input.

**Figure 15:**
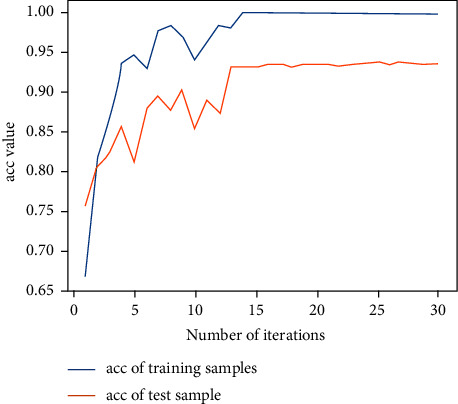
Author identification of the 2D-CNN model under multichannel MNF dimensionality reduced images directly as input.

**Figure 16:**
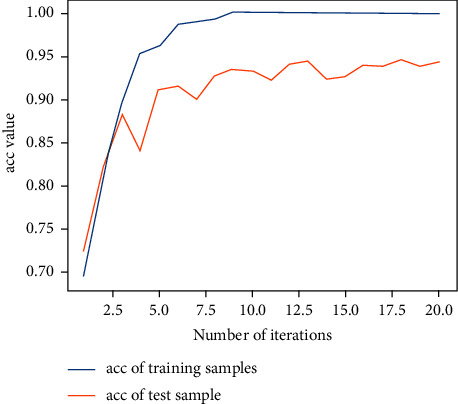
Author identification of the 3D-CNN model under multichannel MNF dimensionality reduced images directly as input.

Next, the previous data are used for training with the 2D-CNN calligraphy and painting identification model under MNF pseudocolor image mosaic as input, 2D-CNN calligraphy and painting identification model under multichannel MNF dimensionality reduced image directly as input, and 3D-CNN calligraphy and painting identification model based on multichannel MNF dimensionality reduced images, and the target outputs are the classification of two authors. The results are shown in Figures 14–16.

It can be seen that the 2D-CNN calligraphy and painting identification model under MNF pseudocolor image mosaic as input, the 2D-CNN calligraphy and painting identification model under multichannel MNF dimensionality reduced image directly as input, and the 3D-CNN calligraphy and painting identification model based on multichannel MNF dimensionality reduced image directly as input all show good convergence. Furthermore, the accuracy of different methods with different kinds of input is counted, respectively, as shown in Table 1.

It can be further seen from Table 1 that for the author identification of different calligraphies and paintings, the accuracy in the test set of the 2D-CNN model under the multichannel MNF images directly as input reaches 93.7%, the accuracy of 2D-CNN based on MNF pseudocolor image mosaic as input reaches 92.6%, and the identification result of 3D-CNN based on multichannel MNF dimensionality reduced images reaches 93.2%. Although the accuracy of the calligraphy and painting author identification model based on 3D-CNN is slightly lower in the computing speed than that of model based on 2D-CNN, its convergence is more stable and faster in the computing step than that of 2D-CNN.

#### 6.4.2. Authenticity Identification Experiment of Different Calligraphies and Paintings

**Figure 17:**
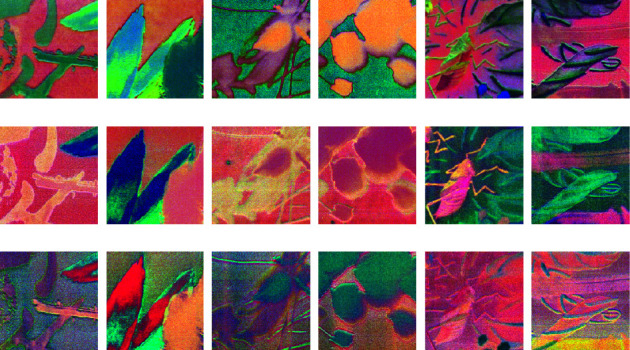
Qi Baishi's true painting's pseudocolor image blocks composed of different channel MNF images. (a) Qi Baishi's true painting's pseudocolor image blocks composed of 1/4/7-th MNF channel images. (b) Qi Baishi's true painting's pseudocolor image blocks composed of 2/5/8-th MNF channel images. (c) Qi Baishi's true painting's pseudocolor image blocks composed of 3/6/9-th MNF channel images.

**Figure 18:**
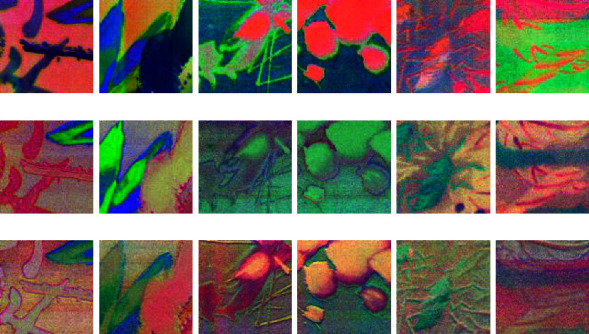
Qi Baishi's fake painting's pseudocolor image blocks composed of different channel MNF images. (a) Qi Baishi's fake painting pseudocolor image blocks composed of 1/4/7-th MNF channel images. (b) Qi Baishi's fake painting pseudocolor image blocks composed of 2/5/8-th MNF channel images. (c) Qi Baishi's fake painting pseudocolor image blocks composed of 3/6/9-th MNF channel images.

In this test, nearly 100 Qi Baishi's true paintings and 30 false paintings are used to perform hyperspectral imaging scanning for obtaining their visible near-infrared hyperspectral image. They are further cut to obtain the hyperspectral image blocks, which actually used for authenticity identification learning of calligraphy and painting. As a result, the amount of training data is also increased to thousands. Then, MNF is performed to obtain the multichannel MNF dimensionality reduced image. For reducing the data amount, the first nine channels MNF images are taken as the last experiment data. Figures 17 and 18 show that it is a group of pseudocolor image blocks composed of different MNF images of true works and fake works. Then, the training of different models was finished according to the previous designation.

It can be seen from the previous figure that the visual information in pseudocolor images synthesized by three channels of MNF images of true paintings and false paintings is obviously different. The spatial textures of the MNF pseudocolor image of Qi Baishi's true and false paintings are basically the same, but there is some difference in the visual pseudocolor, which indicates that MNF images can provide rich feature differences between the true painting and the false painting.

**Figure 19:**
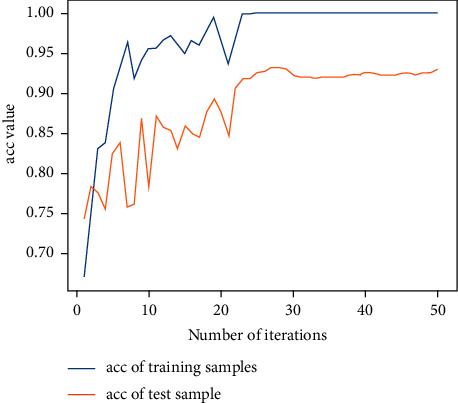
2D-CNN authenticity identification result under MNF pseudocolor images mosaic as input.

**Figure 20:**
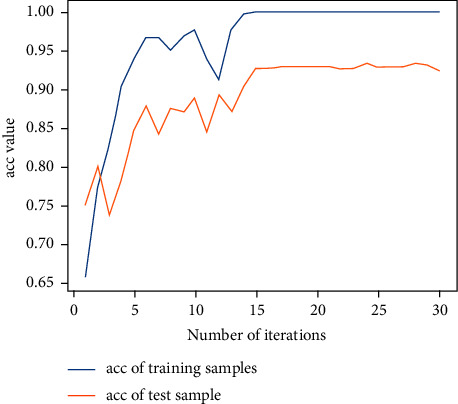
2D-CNN authenticity identification result under multichannel MNF dimensionality reduced images directly as input.

**Figure 21:**
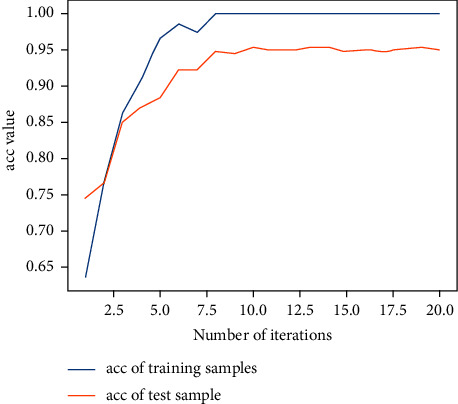
3D-CNN authenticity identification result based on multichannel MNF dimensionality reduced images directly as input.

Next, the previous data are used to train the 2D-CNN calligraphy and painting identification model under MNF pseudocolor image mosaic as input, 2D-CNN calligraphy and painting identification model under multichannel MNF dimensionality reduced image directly as input, and 3D-CNN calligraphy and painting identification model based on multichannel MNF dimensionality reduced images. The results are shown in Figures 19–21.

Similarly, it can be seen that the 2D-CNN calligraphy and painting identification model under MNF pseudocolor image mosaic as input, the 2D-CNN calligraphy and painting identification model under multichannel MNF dimensionality reduced image directly as input, and the 3D-CNN based on multichannel MNF dimensionality reduced images all show good convergence. The accuracy of different models is further counted; the results are shown in Table 2.

It can be further seen from Table 2 that for the authenticity identification of different calligraphies and paintings in the test set, the accuracy of the 2D-CNN calligraphy and painting identification model based on MNF multichannel images directly as input reaches 93.1%, the result of 2D-CNN based on MNF pseudocolor image mosaic as input reaches 92.8%, and the result of 3D-CNN based on multichannel dimensionality reduced images reaches 95.2%. So, the accuracy of 3D-CNN is the best. More importantly, the convergence process of the 3D-CNN calligraphy and painting identification model is more stable and faster than that of the 2D-CNN identification model.

#### 6.4.3. Relevant Failure Cases and Some Discussion

For the painting and calligraphy, when the part of its surface content is colorful, their reflection spectrum information is very rich, and the identification is credible; when the part of its surface content is single black or there is no painting content, their reflection spectrum information is relatively single, and their features are different from the features provided by the calligraphy and painting part with painting content. At this time, in the same work's training, the learning information in different parts with painting content and without painting content is different. Therefore, in the specific training, the hyperspectral image block with painting content is mainly used for training, and the hyperspectral image block without painting content or less content may be wrongly identified.

**Figure 22:**
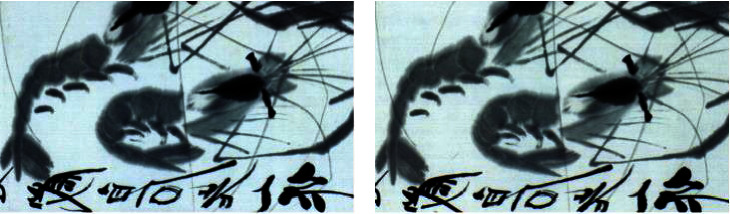
Comparisons of traditional photos of true paintings with ink (a) and false paintings with ink (b).

**Figure 23:**
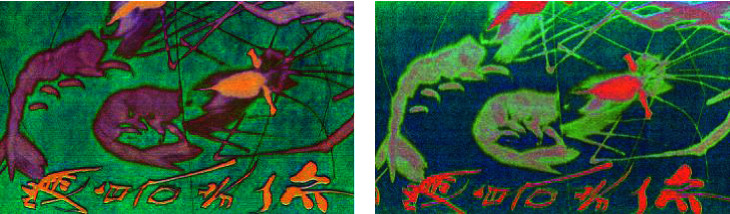
Comparisons of MNF pseudocolor images of true paintings (a) and false paintings with ink (b).

For example, Figures 22 and 23 show that for a group of true paintings and false paintings of Qi Baishi's, when the selected image block includes a lot of single ink information, the classification result judged by the expert eye may be wrong. While in the MNF pseudocolor images of true paintings with ink and false paintings with ink, there is some difference, which could maintain the classification by CNN be feasible.

**Figure 24:**
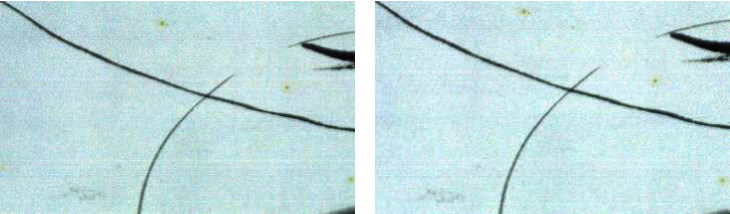
Comparisons of traditional photos of true paintings without color and ink (a) and false paintings without color and ink (b).

**Figure 25:**
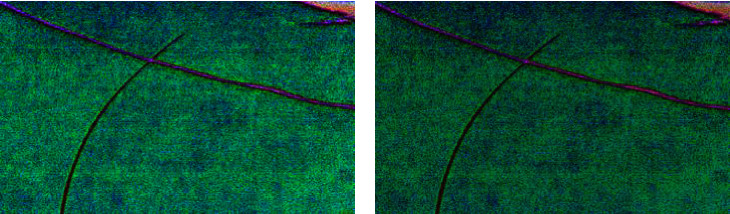
Comparisons of traditional photos of true paintings without color and ink (a) and false paintings without color and ink (b).

However, when there is nothing or a few of information in the painting, the identification result may have a small probability of error. Figures 24 and 25 show that for a group of true paintings and false paintings of Qi Baishi, when the selected image block includes a large part of the paper and a little ink, it is difficult to identify by the expert eye. Meanwhile, there is few different characteristics in the MNF pseudocolor images, which make the judgment wrong sometimes.

In addition, because it is difficult to obtain a variety of true calligraphy and painting samples, in this paper, we only select the calligraphy and painting of Qi Baishi and Lu Yanshao for principal verification. For the identification of a large number of writers, the above learning model needs to be modified appropriately. For example, in calligraphy and painting writers' learning, the number of neurons in the network output layer needs to be adjusted to the number of writers. Meanwhile, in the test verification, a large number of image blocks without direct information are removed, such as the case in Figure 24.

At last, in the training of different kinds of painting, the data with similar characteristic distribution should be selected as much as possible so that the essential difference of calligraphy and painting could be used to make the identification more accurate.

## 7. Summary

By combining the hyperspectral imaging and machine learning, it can greatly improve the comprehension efficiency of calligraphy and painting identification. In order to reduce the redundancy and the amount of network parameters and improve the classification effect in the method of directly using hyperspectral images, we propose a deep learning method of painting and calligraphy identification based on space dimensionality reduction, which is objective convex dimensionality reduction mapping to compress the original hyperspectral image; meanwhile, we use 2D-CNN and 3D-CNN to realize the judgement of the calligrapher/painter and authenticity in the dimensionality reduced image space. The test results show that the 2D-CNN calligraphy and painting recognition model under MNF pseudocolor image mosaic as input and the 2D-CNN calligraphy and painting recognition model under multichannel MNF images directly as input all have high recognition accuracy, while the 3D-CNN calligraphy and painting recognition model based on multichannel MNF dimensionality reduced images not only maintains high recognition accuracy but also improves the convergence speed (step number) and learning stability, compared with the 2D-CNN calligraphy and painting recognition model; in particular, the 2D-CNN calligraphy and painting recognition model for author classification under multichannel MNF images directly as input and the 3D-CNN calligraphy and painting recognition model for authenticity identification based on multichannel MNF dimensionality reduced images perform the best among the methods mentioned in this article. Unfortunately, due to the small number of hyperspectral image samples of calligraphy and painting, especially the small number of fake calligraphy and painting data, the calligraphy and painting recognition model still needs to be further verified and improved.

## Figures and Tables

**Table 1:** Results of calligraphy and painting author identification of different CNN models with different kinds of input.

	*Training set*		
Network model	3D-CNN based on multichannel dimensionality reduced images	2D-CNN based on MNF pseudocolor image mosaic as input	2D-CNN based on multichannel dimensionality reduced images directly as input
Accuracy	1.000	1.000	1.000

	*Test set*		
Network model	3D-CNN based on multichannel dimensionality reduced images	2D-CNN based on MNF pseudocolor image mosaic as input	2D-CNN based on multichannel dimensionality reduced images directly as input
Accuracy	0.932	0.926	0.937

**Table 2:** Authenticity identification results of different calligraphies and paintings based on different CNN models under different inputs.

	*Training set*		
Network model	3D-CNN based on multichannel dimensionality reduced images	2D-CNN based on MNF pseudocolor image mosaic as input	2D-CNN based on multichannel dimensionality reduced images directly as input
Accuracy	1.000	1.000	1.000

	*Test set*		
Network model	3D-CNN based on multichannel dimensionality reduced images	2D-CNN based on MNF pseudocolor image mosaic as input	2D-CNN based on multichannel dimensionality reduced images directly as input
Accuracy	0.952	0.928	0.931

## Data Availability

The part of data used to support the findings of the study can be obtained from the corresponding author upon request.
